# Etymology and the neuron(e)

**DOI:** 10.1093/brain/awz367

**Published:** 2019-12-17

**Authors:** Arpan R Mehta, Puja R Mehta, Stephen P Anderson, Barbara L H MacKinnon, Alastair Compston

**Affiliations:** 1 Centre for Clinical Brain Sciences, University of Edinburgh, Edinburgh, UK; 2 Division of Clinical Neurology, Nuffield Department of Clinical Neurosciences, University of Oxford, Oxford, UK; 3 Department of Basic and Clinical Neuroscience, Maurice Wohl Clinical Neuroscience Institute, King’s College London, London, UK; 4 The National Hospital for Neurology and Neurosurgery, University College London Hospitals NHS Foundation Trust, Queen Square, London, UK; 5 New College, University of Oxford, Oxford, UK; 6 Faculty of Classics, University of Oxford, Oxford, UK; 7 Modern Languages Department, Winchester College, Winchester, UK; 8 Department of Clinical Neurosciences, University of Cambridge, Cambridge, UK

## Abstract

‘Neuron’ or ‘neurone’? While it is often assumed that these different spellings reflect usage of American versus British English, there are also inconsistencies within these cultural boundaries. Mehta *et al.* review historical, etymological and linguistic evidence concerning the spelling of ‘neuron(e)’ and conclude that the only correct spelling is ‘neuron’.

## Introduction

The nerve cell, made up of its axonal appendage and major dendrites, is variously referred to as the ‘neuron’ or ‘neurone’. The reason for preferring one spelling over the other is usually assumed to reflect American (neuron) versus British (neurone) use of the English language. However, the spelling is inconsistent even within these cultural boundaries. For instance, both the Motor Neurone Disease Association (based in the UK) and the USA based International Alliance of ALS/MND Associations refer to ‘motor neurone disease’. Others use the spellings interchangeably, even within the same sentence; see, for example, ‘Mechanism behind neuron death in motor neurone disease and frontotemporal dementia discovered’ (Wellcome, 2018). These agencies are not alone in appearing uncertain as to which is the correct spelling. Attention has previously been drawn to these ambiguities, and opinion expressed on which is the correct spelling (McMenemy, 1963). Here, we trace in more detail the introduction of the word for nerve cell, and provide etymological arguments supporting the view that the correct, and only, spelling is ‘neuron’.

## ‘Neuron’ and ‘neurology’ are derived from classical Greek

Although the term ‘nervous system’ now refers collectively to the brain, spinal cord, and peripheral nerves, with the distinction of central and peripheral added for clarity, the classical Greek word, νεῦρον (neuron), with plural νεῦρα, referred to a plethora of objects—sinew, tendon, gut, and cord in the singular, sinews and nerves in the plural, and sometimes (in its feminine by-form, νευρά) a bowstring—and therefore originally it did not specifically have to do with the nervous systems. Much later, as we show, the word was reintroduced and used to describe the ubiquitous structure made up of the nerve soma and its major appendages present throughout the brain and spinal cord. In his epic poems the *Odyssey* and the *Iliad*, which are amongst the oldest literary sources in Western civilization, Homer (*c.*700/650 BC) used νεῦρα (neura) to indicate the ‘sinews at the top of the leg’ (*Iliad* XVI. 316), and ‘ox sinews’ as a fibre used in making a bowstring (*Iliad* IV. 122). It was probably not until two Hellenistic physicians, Herophilus (*c*.330–*c*.260 BC) and Erasistratus (*c*.325–*c*.250 BC), who moved away from the earlier Aristotelian (384–322 BC) view that the heart is the central organ for action, perception, and cognition (Walshe, 2016) and ascribed these functions to the nervous system, that the structure connecting the brain and spinal cord to sensory organs, the viscera and to muscles was referred to as the νεῦρον (neuron). The Latin ‘nervus’ had a similarly wide range of meanings and is the origin of the word ‘nerve’. Although many important papyrus scrolls describing Greek medicine and anatomy were destroyed in the fire of 391 AD in the great library in Alexandria, we know from the works of Rufus of Ephesus (*c.*80–*c.*150 AD), some 300 years later, that Herophilus used the word νεῦρα (neura) in the context of the anatomy of the nervous system:

‘The nerve is a simple solid body, the cause of voluntary motion, but difficult to perceive in dissection. According to Erasistratus and Herophilus there are nerves capable of sensation, but according to Asclepiades not at all. According to Erasistratus there are two kinds of nerves, sensory and motor nerves; the beginnings of the sensory nerves which are hollow, you can find in the meninges [sc. of the brain], and those of the motor nerves in the cerebrum and the cerebellum. According to Herophilus on the other hand, the *neura* that make voluntary motion possible have their origin in the cerebrum and the spinal marrow, and some grow from bone to bone, others from muscle to muscle, and some also bind together the joints’ (von Staden, 1989).

There is no Greek or Latin word that corresponds exactly to ‘neurology’, a compound term introduced in the 17th century. The model on which this coinage depends is provided by words such as ἀστρολογία (astrologia), μετεωρολογία (meteorologia), φυσιολογία (physiologia), and θεολογία (theologia), where the term’s second element (-λογία/-logia) refers to the body of knowledge on the subject specified by the first. Some of these compound terms were first used by the Greeks; others were introduced later. When the Romans used Greek technical terms, although they sometimes left them in Greek script, they regularly transliterated them, so forming, *inter alia*, their ‘astrologia’ and ‘theologia’.

The prefix ‘neuro-’ dates from Thomas Willis (1621–75) and his two treatises, *Cerebri anatome* etc., and *Nervorum descriptio et usus* etc. (‘The anatomy of the brain’ and ‘The description and use of nerves’), published in Latin and later translated into English (Willis, 1664, 1681). Despite shifts in style during the Reformation, most scholars, including physicians, were slow to adopt the vernacular and continued to write in Latin. This was the language in which the treatises of Thomas Willis were all first printed (apart from *A plain and easie method for preserving those that are well from the plague*, published posthumously in 1691), although several words in each treatise were set using Greek typeface. Willis’s occasional use of Greek coinage, rather than Latin, was consistent with the practice of learned physicians and scholars of all types from the Renaissance onwards; and some Roman writers, such as Cicero, quite regularly used Greek expressions when writing in Latin.

**Figure 1:**
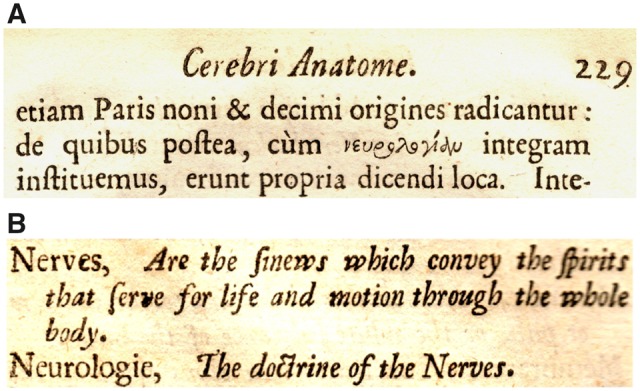
**Thomas Willis (1621–75) and the introduction of the prefix ‘neuro-’ into medical terminology.** (**A**) Text from *Cerebri Anatome: cui accessit Nervorum Descriptio et Usus* (1664, p. 229) in which Thomas Willis first sets out his intention to discuss ‘neurologie’. (**B**) The definitions of ‘nerve’ and ‘neurologie’ used by Samuel Pordage in the table of hard words appended to his translation of Willis’s two treatises, *The Anatomy of the Brain* and *The Description and use of the Nerves* (1681): from original copies of each (Willis, 1664, 1681).

‘Nευρολογίας pensum, difficile licet, utile ac iucundum est, 235.’ (‘The task of Neurology [or ‘the Doctrine of the Nerves’], though difficult, is useful and pleasing.’) is first printed in the ‘Elenchus rerum’ of the 1664 4to edition of *Cerebri anatome*. It refers the reader to page 235, where the text reads: ‘Idcirco, etiamnum velis vento datis, procedere, & difficile νευρολογίας pensum aggredi statuimus’. In his English translation (1681), Samuel Pordage (1633–91), a poet who also styled himself ‘a student of physick’, has this (at page 125) as: ‘Therefore although we know it is difficult to proceed with full Sail, we have resolved to undertake the task of the Doctrine of the Nerves.’ (Pordage’s translations were notoriously imprecise and a preferred wording might be: ‘Therefore, even now spreading our sails to the winds, we have resolved to proceed and to undertake the task of the Doctrine of the Nerves’). In fact, although not listed in the ‘Elenchus rerum’, Willis had first used νευρολογία at page 229: ‘de quibus postea, cum νευρολογίαν integram instituemus, erunt propria dicendi loca’ (Fig. 1A). At page 123, Pordage translates this as: ‘Of which there will be hereafter a proper place to speak, when we shall institute the whole Neurology or the Doctrine of the Nerves’. And in his table of hard words, Pordage defines ‘Nerves’ as: ‘*the sinews which convey the spirits that serve for life and motion through the whole body*’; and ‘Neurologie’ as ‘*The doctrine of the Nerves*’ (Fig. 1B).

## ‘Neuron’: first English appearance

**Figure 2:**
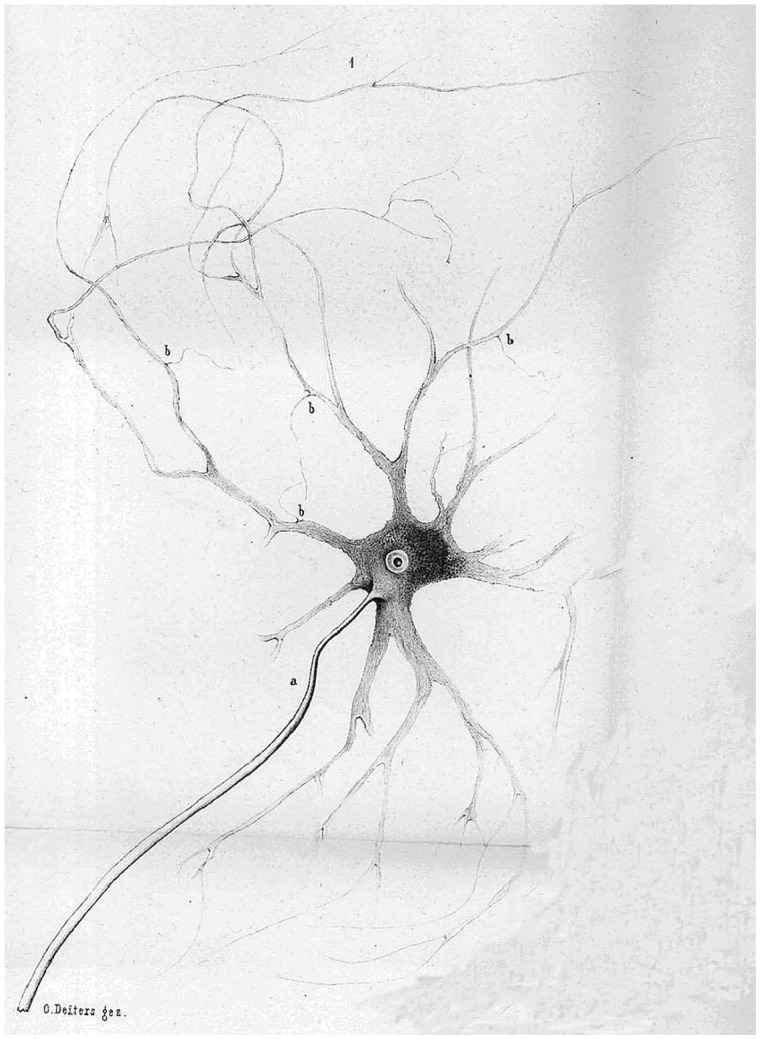
**The first drawing of a neuron as the nerve cell and its processes.** These were published in 1865, in posthumous work by Otto Friedrich Karl Deiters (1834–63). In the centre, he depicts the cell body with its nucleus; (b) represents the multiple dendrites and (a) represents the single axon (Deiters, 1865).

**Figure 3:**
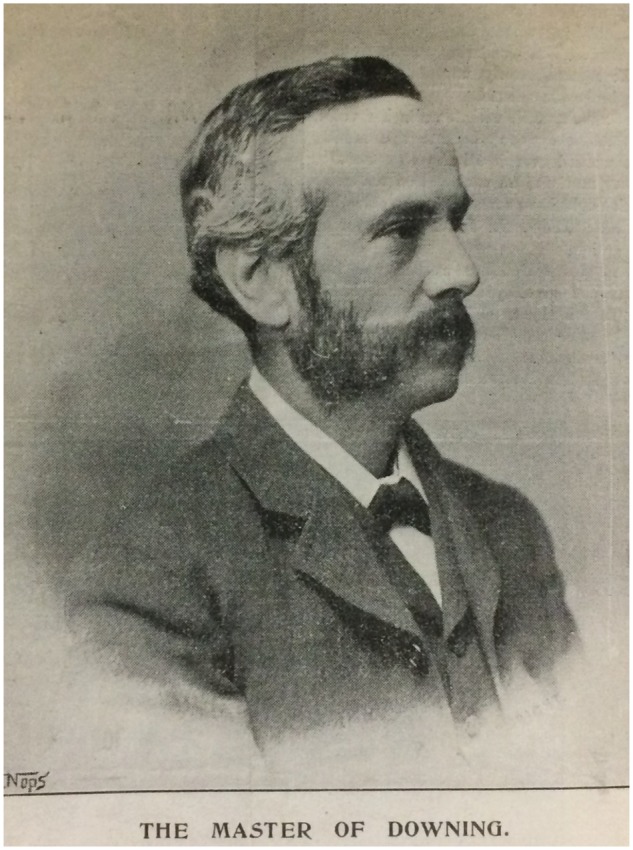
**Alexander Hill (1856–1929), anatomist and surgeon, and Master of Downing College, Cambridge (1888–1907).** Sadly, little is known about his academic, medical or personal life, and it remains a mystery how and why he was involved in neurology. Image courtesy of Downing College Archives, originating from a book of press cuttings from *The Times*, 2 February 1902, about his appointment as Bursar (ref. DCPP/STE/1/1).

**Figure 4:**
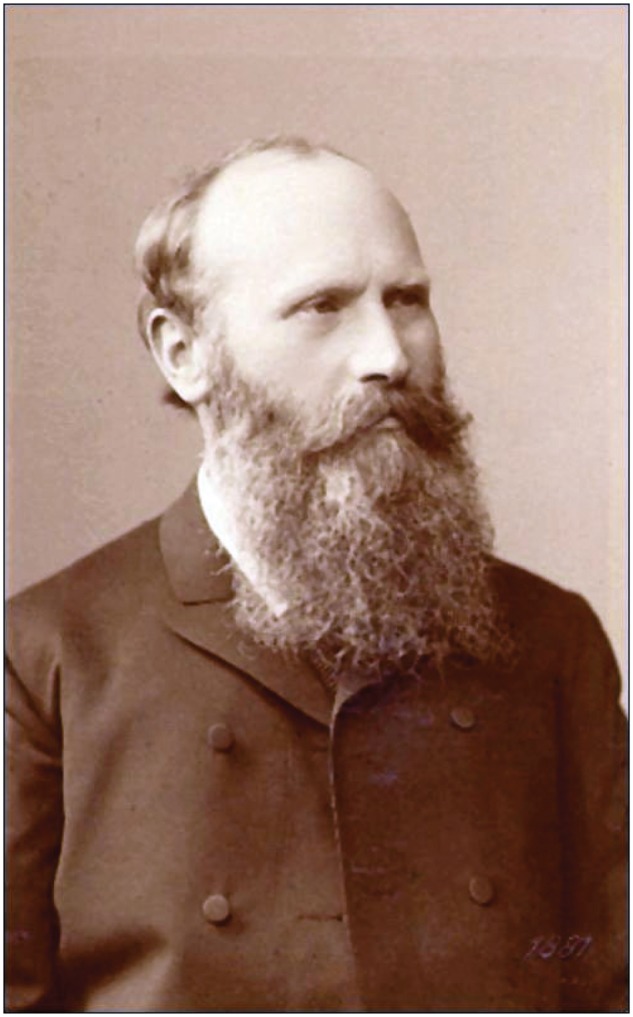
**Heinrich Waldeyer (1836–1921).** Born into a family of aristocratic extraction and originally intending to study music and mathematics, Waldeyer was attracted to medicine and, after professorships in Breslau and Strasbourg, he worked in Berlin on human and comparative anatomy earning a reputation, by 1891, as a scientist, administrator and public figure (Shepherd, 1991).

After an interval of more than two millennia, the concept of the ‘neuron’ was reintroduced, probably in ignorance of its former meaning, but it took time for general agreement to be reached on what the term now defined. The Oxford English Dictionary attributes first use of the English term ‘neuron’ to a paper by Benjamin Thompson Lowne (1839–1925), where it denotes the neural part of the compound eye of arthropods (Lowne, 1883). The first neurologist to use the term was an American, Burt Green Wilder (1841–1925), who had a penchant for neurological nomenclature (which he termed ‘neuronymy’). In his Cartwright Lectures of 1884, Wilder used ‘neuron’ to describe the whole neuraxis (Wilder, 1896). However, first use of the term in describing the nerve cell and its processes (illustrated as such by Deiters; Fig. 2) was by the English anatomist and physician, Alexander Hill (1856–1929; Fig. 3) who, in 1891, published in *Brain* a translation of the German paper based on the lectures of Heinrich Wilhelm Gottfried von Waldeyer-Hartz (1836–1921; Fig. 4) to the Berlin Medical Society (Hill, 1891). Waldeyer coined the term ‘die Neuronen’ (singular ‘das Neuron’), as an alternative for ‘Nerveneinheiten’ or ‘nerve units’:

‘4. Somit besteht ein Nervenelement (eine „Nerveneinheit“ oder „Neuron“, wie ich es zu nennen vorschlagen möchte), den genannten Forschungsergebnissen (wenn wir einen netzartigen Zusammenhang nicht gelten lassen) zufolge, aus nachstehenden Stücken: a) einer Nervenzelle, b) dem Nervenfortsatze, c) dessen Collateralen und d) den Endbäumchen. Diese einfachste Form des Neurons scheint in der That bei den höheren Evertebraten (untersucht wurden Kruster und höhere Würmer) fast ausschliesslich vorzukommen (Nansen, G. Retzius, Biedermann)’ (Waldeyer, 1891).

This was translated in Alexander Hill’s paper:

‘4. Thus a nerve element, a nerve entity, or ‘neuron’, as I propose to call it, consists as the results of observation show (if we do not allow the existence of a reticular connection) of the following pieces:–(*a*) a nerve cell, (*b*) the nerve process, (*c*) its collaterals, and (*d*) the end-branching. This simplest form of the neuron appears, in fact, to be exclusively present in the higher invertebrates as investigated by Nansen, G. Retzius, Biedermann’ (Hill, 1891).

Until the early 1890s, in describing the structure designated ‘neuron’ by Waldeyer, all commentators referred to the nerve or ganglion cell, and nerve fibres, processes or cylinders in English or equivalent terms in French and German. It is said that Santiago Ramón y Cajal (1852–1934), his contemporary, wrote that all Waldeyer had done was to ‘publish in a daily paper a résumé of Cajal's research and invent the term neuron’. However, Waldeyer must have had a sensitivity for words given that, 3 years earlier, he had also coined the term ‘chromosome’. Gordon Shepherd states:

‘William Waldeyer … summarised the new findings [of Golgi and Cajal] in a coherent theory, which stated that *the nerve cell is the anatomical, physiological, metabolic, and genetic unit of the nervous system*. To emphasise the newly recognised character of the nerve cell, Waldeyer bestowed on it a new name, the *neuron*. This formulation of the cell theory in terms of the specific types of cells found in the nervous system came to be called the *neuron doctrine*’ (Shepherd, 1991).

Waldeyer’s proposed terminology was soon adopted by others, although Sir Edward Sharpey-Schafer (1850–1935) caused temporary confusion by suggesting that only the axonal process of the nerve cell should be designated the ‘neuron’, and the simpler term, ‘nerve-cell’, used ‘as is done for every cell of the body’ (Schäfer, 1893).

By way of example, writing in the early 1890s, Sir Charles Sherrington (1857–1952) referred to ‘nerve cells’ but, by 1897, he was using the terms ‘neuraxon’ and ‘neuron’. [His text included in Foster (1897) on page 929 is doubly important in showing not only his (unattributed) switch to Waldeyer’s nomenclature but also the introduction of the term ‘synapsis’ which, as explained in a footnote, is ‘From σύν and ἅπτω clasp’.]

In revising the first volume (all published) of his *Manual of diseases of the nervous system* for a third edition, at page 55 Sir William Gowers (1845–1915) explained that ‘the name “neuron” proposed by Waldeyer has been all but universally adopted … its plural is formed according to the living language, and not the classical form–in English it is “neurons”, in German “[N]euronen”, in French “neurones”’. Gowers adds in a footnote:

‘Because the term “axon” is the separate “nerve”, when one exists, an attempt has been made to make current the use of “neuron” for this alone. But etymological consistency has little influence on the vitality of names. The use of “neuron” for the whole element has become so general that resistance to it is futile. Moreover, the conception attached to it in use is already definitely detached from its etymology. Lastly, although the cell-body and its processes are one, to have only one word “cell” for the whole element, a word that will still, inevitably, be applied to the cell body, leaves the latter without nominal distinction from the other two parts of the element–the neuron and dendrons. Hence the word “neuron” is here used in the established senses’ (Gowers, 1899).

But even though anatomical precision was achieved, the confusion on spelling soon re-emerged. Sir Frederick Mott (1853–1926) entitled his Croonian lectures delivered to the Royal College of Physicians on 19, 21, 26 and 28 June 1900 ‘The degeneration of the neurone’. He rehearsed Gowers’s position, explaining that the term ‘neurone’ was introduced by Waldeyer for ‘the nerve cell and all its processes, including the protoplasmic processes or dendrons and the single axis-cylinder process with its cone of origin, its collaterals or side branches, and its terminal arborisation’.

## ‘Neurone’: first appearance in French

Arthur Van Gehuchten (1861–1914), a Belgian anatomist and neurologist, adopted Waldeyer’s coinage, but spelt this in French as ‘le neurone’ (Van Gehuchten, 1893). This was also the spelling used in Spain and Italy (Barker, 1896). We believe that the reason for adding the ‘e’ at the end of the word relates to the interplay between linguistics and phonetics: the final ‘n’ in ‘neuron’ would have been ‘sounded’ in the classical Greek, and also in Waldeyer’s German coinage, and, to do the same in French, there needed to be an ‘e’ placed at the end of the word. Without this, ‘neuron’ would have rhymed with ‘maison’ and the link with the original Greek would have been lost. There are other examples of this, such as ‘Babylon’ being spelt ‘Babylone’ in French. Hill, as described above, had already anglicized Waldeyer’s coinage as ‘neuron’ and so there appears no justification for transferring this to English through use of the French, ‘neurone’.

## Etymological flaws

Uncertainty with respect to the spelling of ‘neuron(e)’ persisted and without respecting rigorous geographical or cultural boundaries. The Canadian neurologist, Lewellys F. Barker (1867–1943), who succeeded Sir William Osler (1849–1919) as physician-in-chief at Johns Hopkins Hospital in 1905, used the term ‘neurone’ in his textbook, *The Nervous System* (Barker, 1899). Here, he states that the Greek word from which Waldeyer coined the term is νευρών. If accepted, Sir William Bayliss (1860–1924) suggested that, in order to ensure a long *o* in pronunciation, it must be spelt in English with the final *e*; however, nowhere in Waldeyer's paper does he mention the Greek word from which the term was adopted (Bayliss, 1916). Moreover, Bayliss comments that Sir Charles Sherrington had pointed out that νευρών does not exist in classical Greek. Nor is it to be found interpolated into classical Greek dictionaries. The correct singular usage is νεῦρον, plural νεῦρα; and there is also the cognate feminine form νευρά, plural νευραί. Both words produce a genitive plural, in unaccented form νευρων and, with accents added, νεύρων and νευρῶν, respectively. Each is spelt the same as the non-existent Greek word, but with different accentuations.

Finally, Basil Lanneau Gildersleeve (1831–1924), an American classical scholar, was consulted by Barker and it is informative to revisit and amplify his analysis (Barker, 1896). The words, ‘anode’ and ‘cathode’ derive from the Greek words, ἄνοδος and κάθοδος, meaning, literally, ‘way up’ and ‘way down’. These are both extensions of the word ὁδός, which means ‘road’ or ‘way’. There is, however, no reason why they must produce in the English ‘anode’ and ‘cathode’ (with an ‘e’), as the word μέθοδος, also a compound of ὁδός, gives simply ‘method’. Similarly, νεῦρον, should be ‘neuron’, in the same manner that ‘proton’ is derived from πρῶτον. Furthermore, even if the origin of the Greek word was νευρών (which we argue does not exist, at least as a nominative), there still is no requirement for an ‘e’ at the end in the English given that, for example, ‘Parthenon’ (meaning ‘maiden’s house’) is derived from the Greek word Παρθενών, and it is never spelt as ‘Parthenone’.

## Recommendation

The present acceptance of two spellings is understandable, given the ambiguities created at a critical time in the shaping of 19th and 20th century neurology. Nevertheless, it is clear from multiple levels of evidence (historical, etymological and linguistic) that the only correct spelling for the structure made up of the nerve soma, axon, and some dendrites is ‘neuron’, pronounced ‘nyuor-ron’ (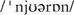 in English Received Pronunciation). Our position is that ‘neurone’, in any medical or cultural context, should no longer be used.
